# The contribution of lower-limb joint quasi-stiffness to theoretical leg stiffness during level, uphill and downhill running at different speeds

**DOI:** 10.1098/rsos.231133

**Published:** 2024-04-17

**Authors:** Caelyn E. Hirschman, Jana R. Montgomery, Alena M. Grabowski

**Affiliations:** ^1^ Applied Biomechanics Lab, University of Colorado Boulder, Boulder, CO, USA; ^2^ VA Eastern Colorado Healthcare System, Denver, CO, USA

**Keywords:** sloped running, spring–mass model, biomimetic device design, biomechanics

## Abstract

Humans change joint quasi-stiffness (*k*_*joint*_) and leg stiffness (k_leg_) when running at different speeds on level ground and during uphill and downhill running. These mechanical properties can inform device designs for running such as footwear, exoskeletons and prostheses. We measured kinetics and kinematics from 17 runners (10 M; 7 F) at three speeds on 0°, ±2°, ±4° and ±6° slopes. We calculated ankle and knee *k*_*joint*_, the quotient of change in joint moment and angular displacement, and theoretical leg stiffness (k_legT_) based on the joint external moment arms and *k*_*joint*_. Runners increased *k*_*ankle*_ at faster speeds (*p* < 0.01). Runners increased and decreased the ankle and knee contributions to k_legT_, respectively, by 2.89% per 1° steeper uphill slope (*p* < 0.01) during the first half of stance. Runners decreased and increased ankle and knee joint contributions to k_legT_, respectively, by 3.68% during the first half and 0.86% during the second half of stance per 1° steeper downhill slope (*p* < 0.01). Thus, biomimetic devices require stiffer *k*_*ankle*_ for faster speeds, and greater ankle contributions and greater knee contributions to k_legT_ during the first half of stance for steeper uphill and downhill slopes, respectively.

## 1. Introduction

The function of passive-elastic biomimetic assistive device designs used for running, such as lower limb prostheses and exoskeletons, is based on specific mechanical parameters such as stiffness. Determining how lower limb joint quasi-stiffness changes and how lower limb joints contribute to leg stiffness could inform and improve assistive device design (i.e. running-specific leg prostheses) for running on slopes and level ground. The stance phase mechanics of level-ground steady-speed running in animals, including humans, are well described by a spring–mass model, where the leg is represented as a massless linear spring and body mass is represented as a point mass that indicates the centre of mass (COM) [1–4]. Leg stiffness equals the quotient of peak vertical ground reaction force (GRF) and leg spring displacement [4]. Humans adjust leg stiffness to accommodate changes in terrain [3,5,6] and may increase leg stiffness when running at faster speeds from 3 m s^−1^ up to maximum speed on level ground [7,8]. These adjustments in leg stiffness are achieved by changing leg muscle force and leg segment position, which can alter joint quasi-stiffness through changes in joint moments and/or limb segment angles [9]. For example, greater leg stiffness can be realized by reducing the change in limb segment angles and thereby decreasing leg displacement for a given force during the stance phase of level-ground running [10]. Moreover, the function elicited by lower limb passive-elastic device designs used for running, such as footwear, exoskeletons and prostheses, which may or may not allow altered leg segment position, depends on specific mechanical parameters such as stiffness. Determining how lower limb joint quasi-stiffness changes and how the lower limb joints contribute to leg stiffness in biological legs during running on level ground, and uphill and downhill slopes could inform and improve such biomimetic device designs.

Previous studies have used a lower extremity rigid segment model to characterize the spring-like mechanics of the ankle and knee joints during the stance phase of level-ground running [1–4]. The ankle and knee joints have been characterized as rotational springs with angular stiffness. The angle–moment relationship of the ankle and knee joints during running can be described as joint quasi-stiffness due to the presence of active (muscle) and passive (tendon and ligament) components that contribute to the overall angle–moment relationship or net external mechanical behaviour [11,12]. Angular joint quasi-stiffness equals the quotient of the change in joint moment and angular displacement, which is often calculated during the first half of the stance phase or from touch down to mid-stance [9,13,14]. Runners may modify lower extremity joint quasi-stiffness when running at faster speeds on level ground, where the ankle and knee joint quasi-stiffness increase by ~73 and ~68%, respectively, during the first half of the stance phase from 1.8 to 3.8 m s^−1^ [14]. These increases in joint quasi-stiffness likely result from greater peak joint moments with little change in angular displacement [14] and relate to an increase in overall leg stiffness [9].

Running indoors or outdoors may require the negotiation of level-ground, uphill and downhill slopes. During uphill and downhill running, leg stiffness differs during the first and second half of the the stance phase [15]. A previous study investigated the ankle and knee joint quasi-stiffness when running at 3 m s^−1^ on level ground and slopes [16] and found that during the first half of the stance phase, ankle joint quasi-stiffness decreased when running −5° downhill compared with level ground and knee joint quasi-stiffness increased when running +5° uphill compared with level ground. To our knowledge, no previous studies have investigated the contribution of the lower limb joints to leg stiffness when running uphill and downhill. However, previous studies have analysed joint mechanical work, which equals the product of the joint moment and angle, during the stance phase of running on slopes. Mechanical work done at each joint during running or walking can inform the design of powered biomimetic devices and be used to predict joint quasi-stiffness changes on slopes [17,18]. Ankle and knee joint mechanical work does not change when running uphill compared with level ground at 3.0−3.5 m s^−1^ [19], but the ankle and knee extensor muscles absorb a greater magnitude of negative mechanical work (13.5 and 28 J, respectively) during the first half of stance when running downhill compared with level ground at 4.5 m s^−1^ [17]. Thus, ankle and knee joint quasi-stiffness may not change during uphill compared with level-ground running during the first and second half of stance but may be stiffer during the first half of stance and less stiff during the second half of stance during downhill compared with level-ground running. Further, Nuckols *et al*. [18] found that when running on a range of uphill and downhill slopes (0˚, ±2.86° and ±5.71° at 2.25 m s^−1^), the muscles surrounding the ankle contributed the most (47−55%) net positive mechanical power to the COM compared with the knee and hip joints, where net mechanical work equals the integral of net mechanical power with respect to time. These results suggest that during running on uphill and downhill slopes, the contribution of the ankle joint to leg stiffness may be greater than the contribution of the knee joint to leg stiffness compared with level-ground running.

Directly relating the contribution of the function of individual joints to leg stiffness could facilitate a better understanding of the underlying biomechanics of running on level ground and slopes at different speeds. Leg stiffness, which is a linear measure with units of N m^−1^, depends on the quotient of the squared external moment arm of each joint and joint quasi-stiffness, which is an angular measure with units of N m rad^−1^ [9]. Moholkar [9] measured the average external moment arm (R_joint_) of the ankle and knee joints and angular ankle and knee joint quasi-stiffness (*k*_*joint*_) in N m rad^−1^ during the first half of stance during level-ground running to calculate ‘theoretical’ leg stiffness values (k_legT_) in N m^−1^ using a novel equation [9]. In this equation, the compliance of k_legT_ (1/k_legT_) equals the sum of R_joint_^2^/*k*_*joint*_ for the ankle and knee joints. On average, theoretical leg stiffness during the first half of stance was within 10.7% (1.7 kN m^−1^) of leg stiffness, which was calculated from the slope of the force–leg displacement curve during level-ground running at 2.5 m s^−1^ [4,9]. The equation presented by Moholkar [9] excludes the hip joint because maximum hip joint angular displacement does not occur within 10% of mid-stance as the hip extends throughout the stance phase and does not act as an angular spring. This is not to say that the hip does not contribute to overall leg stiffness, it likely does, especially at steeper uphill slopes where the external moment arm is larger compared with level ground, but it cannot be included in the equation by Moholkar [9,19]. Though theoretical leg stiffness did not equal calculated leg stiffness or include the hip joint, the theoretical leg stiffness equation provides a framework to determine the ankle and knee joint contributions to leg stiffness during running.

Passive-elastic biomimetic device designs for running are based on the spring-like function of the biological joints and legs. Thus, determining joint quasi-stiffness and leg stiffness during running on uphill and downhill slopes would inform such device designs. Furthermore, determining and comparing ankle and knee joint quasi-stiffness values during the first and second half of stance can infer if these joints provide spring-like function. If quasi-stiffness values differ during the first compared with the second half of the stance phase, this may indicate that the joint does not behave like a perfect spring. However, passive-elastic device designs rely on stiffness values to provide spring-like function to the user and thus we aimed to determine discrete joint quasi-stiffness values to inform these device designs. Previous studies have shown that passive-elastic device designs are able to adjust the stiffness response and timing during the stance phase of walking and across different modes of locomotion [20–23], thus our results could be used in future studies aimed at mimicking the biological ankle and knee during running and to inform passive-elastic device designs.

We determined ankle and knee joint quasi-stiffness and theoretical leg stiffness during running at different speeds on uphill and downhill slopes. First, we hypothesized that when running faster regardless of slope, ankle joint quasi-stiffness and theoretical leg stiffness would increase during the first and second half of stance, and the contribution of the ankle and knee joints to theoretical leg stiffness would not change during the first and second half of stance. Second, we hypothesized that when running on a steeper uphill slope regardless of speed, knee joint quasi-stiffness and theoretical leg stiffness would increase during the first half of stance, ankle joint quasi-stiffness and theoretical leg stiffness would increase during the second half of stance, and the contribution of the ankle and knee joints to theoretical leg stiffness would increase and decrease, respectively, during the second half of stance. Finally, we hypothesized that when running on a steeper downhill slope regardless of speed, ankle joint quasi-stiffness and theoretical leg stiffness would decrease during the first and second half of stance, and the contribution of the ankle and knee joints to theoretical leg stiffness would decrease and increase, respectively, during the first half of stance.

## 2. Methods

### 2.1. Participants and protocol

Seventeen healthy human runners (10 males, 7 females; mean ± s.d.: age 26.6 ± 6.2 years, mass 68.7 ± 8.4 kg and height 1.76 ± 0.09 m) participated and provided written informed consent according to the Department of Veterans Affairs Eastern Colorado Healthcare System Human Subjects Institutional Review Board approved protocol (COMIRB #12-0553). Subjects reported no cardiovascular, pulmonary or neurological disease or disorder, and no musculoskeletal injuries. Each subject performed 21 running trials that included three speeds on 0°, ±2°, ±4° and ±6° slopes (table 1) while we measured their kinematic and kinetic data. These data were collected as part of a larger study that included measurements of metabolic rates, data not reported in the present study. To ensure that each subject could run using primarily aerobic metabolism, running speeds on uphill slopes, which ranged from 1.5 to 2.75 m s^−1^ differed from those on level ground and downhill slopes, which ranged from 2.25 to 3.0 m s^−1^ (table 1). Running trials were performed over 4 days. During the first 3 days, we measured metabolic rates during 5 min trials for each speed and slope. During the 4th day, we measured kinematic and kinetic data for 30 s during each ~1 min trial for each speed and slope.

**Table 1.** Subjects completed 21 running trials at different speeds and slopes.

slope	speed (m s^−1^)		
	slow	medium	fast
−6°, −4°, −2°, 0°	2.25	2.5	3.0
+2°	2.25	2.5	2.75
+4°	1.75	2.0	2.5
+6°	1.5	1.75	2.0

### 2.2. Biomechanical measurements

Prior to running trials on the fourth day, we used a modified Helen Hayes marker set and placed 30 reflective markers bilaterally on the lower extremity over the ankle, knee and hip joint centres and in clusters on each lower limb segment including the pelvis. Then, we captured a static standing trial on level ground for each subject to create a 6DOF lower-body rigid segment model using Visual 3D (C-Motion, Germantown, MD, USA). Subsequently, each subject performed 21 running trials in a randomized order (table 1) on a force-measuring treadmill (Treadmetrix, Park City, UT, USA). Each trial was at least 1 min long with ad libitum rest between trials. We simultaneously collected three-dimensional kinematic data at 200 Hz using a 10-camera motion capture system (Vicon, Centennial, CO, USA) and three-dimensional GRF data at 1000 Hz using the force-measuring treadmill for 30 s. Motion capture marker trajectories were tracked in Vicon and exported to Visual 3D. Kinematic and kinetic data were filtered using a fourth order low-pass Butterworth filter with a 15 Hz cutoff [24]. We constructed a 6DOF lower-body rigid segment model in Visual 3D and the model was scaled by body mass and height for each participant. We used a 20 N perpendicular GRF threshold to determine ground contact and analysed at least 15 steps from each leg per trial. A step was defined as ground contact and the subsequent aerial phase for the same leg [25]. All steps were resampled to 101 data frames and represented as a percentage.

Sagittal plane ankle, knee and hip joint angles and moments were calculated via Visual 3D’s compute model-based data function (C-Motion). The ankle joint angle refers to the angle between the foot and shank segments, where 0 rad is defined by the static position and positive and negative ankle angles indicate dorsiflexion and plantarflexion, respectively. The knee joint angle refers to the angle between the shank and thigh segments, where 0 rad is defined by the static position and positive and negative knee angles indicate flexion and extension, respectively. The hip joint angle refers to the angle between the thigh and pelvis segments, where 0 rad is defined by the static position and positive and negative hip angles indicate extension and flexion, respectively. We report joint angles in radians and use radians to calculate joint quasi-stiffness using radians. We report and set the slope of the treadmill in degrees, rather than radians, as it may be more intuitive.

The GRF and ankle, knee and hip joint angle and moment data were processed using a custom MATLAB script (MathWorks, Natick, MA, USA). We normalized moments by body mass to be consistent with Moholkar [9] and reduce potential variability. We calculated the ankle and knee joint external moment arms and ankle and knee joint quasi-stiffness during the first and second half of the stance phase. We excluded the hip external moment arm and hip joint quasi-stiffness from our calculations because the hip does not act like a spring during level-ground and sloped running [9,19]. The external moment arms for the ankle and knee joints were calculated using a custom MATLAB script and equal the average perpendicular distance from the resultant GRF vector to the joint centre of rotation during the initial first half of stance (20% of peak moment (*M*_peak_) to maximum joint angular displacement during the first half of stance) and the final second half of stance (maximum angular displacement to 20% of *M*_peak_ during the second half of stance). We excluded the initial and final 20% of *M*_peak_ owing to the inherent centre of pressure noise at the beginning and end of the stance phase [26]. We calculated discrete values of ankle and knee joint quasi-stiffness, denoted as *k*_*jointI*_, during the first (initial) half of the stance phase and rebound joint quasi-stiffness, denoted as *k*_*jointR*_, during the second (final) half of the stance phase based on a previous study that suggests leg stiffness may differ between the first and second half of the stance phase when running on slopes [15] (equation (2.1)). We based this distinction on previous literature that suggested use of such terminology when quantifying quasi-stiffness of the biological joints or leg during the second half of the stance phase of locomotion [11].


(2.1) $$k_{joint}=\Delta M/\Delta \theta .$$

We calculated discrete quasi-stiffness values for the ankle and knee joints [9]. We purposely use italics to indicate *angular* joint quasi-stiffness. Initial *k*_*jointI*_ at the ankle and knee equals the change in moment (Δ*M*) divided by the change in angle (Δ*θ*), where we calculated Δ*M* and Δ*θ* from 20% of *M*_peak_ to the moment and angle at maximum joint angular displacement. Final *k*_*jointR*_ equals the quotient of Δ*M* and Δ*θ* from 20% of *M*_peak_ to the moment and angle at maximum angular displacement during the second half of stance. Joint external moment arms and joint quasi-stiffness were calculated and averaged from the left and right legs across at least 15 steps.

To determine the contributions of the ankle and knee joints to overall theoretical leg stiffness, we calculated initial theoretical leg stiffness (k_legTI_) and final rebound theoretical leg stiffness (k_legTR_) using the average external moment arm (R_joint_) and joint quasi-stiffness (*k*_*joint*_) of the ankle and knee according to Moholkar [9, appendix A] (equation (2.2)).


(2.2) $$k_{legT}=\frac{1}{\frac{R_{ankle^{2}}}{k_{ankle}}+\frac{R_{knee^{2}}}{k_{knee}}}.$$

To calculate initial k_legTI_, we used the average initial R_joint_ and *k*_*jointI*_ for the ankle and knee during the first half of stance. To calculate the final k_legTR_, we used the average final R_joint_ and *k*_*jointR*_ for the ankle and knee during the second half of stance.

Then, to determine the contributions of the ankle and knee joints to theoretical leg stiffness we rearranged equation (2.2) and set it equal to 1 (equation (2.3)). We calculated each joint’s contribution to k_legT_ as the joint’s moment arm squared divided by that joint’s stiffness (R_joint_^2^/*k*_*joint*_) multiplied by k_legT_.


(2.3) $$1=\left(\frac{R_{{ankle}^{2}}}{k_{ankle}}\right)\times k_{legT}+\left(\frac{R_{knee}^{2}}{k_{knee}}\right)\times k_{legT}.$$

We did not include the hip joint in equation (2.2) or equation (2.3) because maximum hip joint angular displacement does not occur within 10% of mid-stance [9]. Additionally, the hip has a small external moment arm (1.0–2.9 cm) at the slopes we tested and does not behave like a spring during the stance phase of running (electronic supplementary material, table S1). Unlike the ankle and knee joints, which flex during the first half of stance and extend during the second half of stance, the hip joint almost exclusively extends throughout stance, which means that initial joint quasi-stiffness and final rebound joint quasi-stiffness cannot be calculated for the hip joint (electronic supplementary material, figure S1).

### 2.3. Statistical analysis

We used two linear mixed models [27] to determine whether speed (m s^−1^) or slope (degrees) had a main or interaction effect on *k*_*joint*_ (N m kg^−1^ rad^−1^), k_legT_ (N m^−1^ kg^−1^) or the ankle and knee joint contributions (%) to k_legT_ (*p* < 0.05). We used one model for uphill slopes and one model for downhill slopes. Within the models, the fixed effects were speed (continuous), uphill slope (continuous) and downhill slope (continuous), respectively, and subject was a random effect. We report the fixed effect (*B*) of the main effect or interaction effect of speed or slope on initial and final *k*_*joint*_, k_legT_ and the ankle and knee joint contributions to k_legT_ (equation (2.3)) for each significant association (dependent variable = *B* × independent variable + intercept). We set significance at *p* < 0.05 and performed all statistical analyses using R-studio software (Boston, MA, USA). Due to the lack of overlapping speeds at +4**°** and +6**°** compared with 0**°** and +2**°** (table 1), we did not determine interaction effects for uphill slopes.

## 3. Results

### 3.1. Main effect of speed

**Figure 1.**
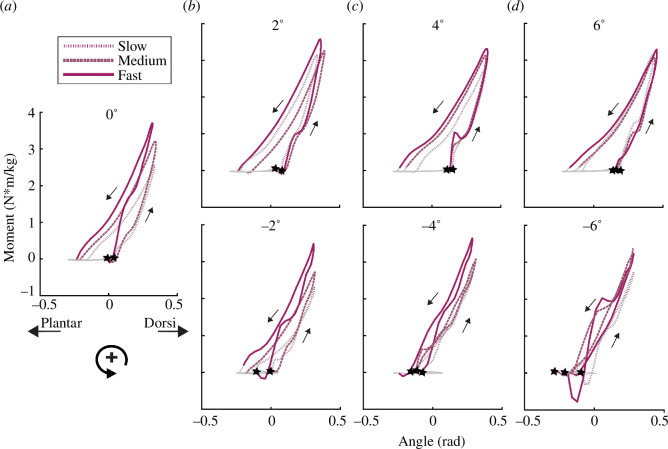
Ankle joint moment versus angle for a step during running at (*a*) 0°, (*b*) ±2°, (*c*) ±4° and (*d*) ±6° at slow, medium and fast speeds (table 1) for a representative subject. The slope of the line (quotient of change in moment and change in angle) from 20% of the peak joint moment (*M*_peak_) to maximum joint angular displacement represents initial *k*_*ankleI*_ and from maximum joint angular displacement to 20% of *M*_peak_ represents the final *k*_*ankleR*_. Positive ankle angle indicates dorsiflexion (Dorsi) and negative ankle angle indicates plantarflexion (Plantar). ⋆ indicates foot contact. The counterclockwise work loop indicates net positive joint mechanical work and the area inside the loop represents the magnitude of work. The grey lines represent the swing phase and the coloured lines represent the stance phase for uphill, level and downhill slopes.

**Figure 2.**
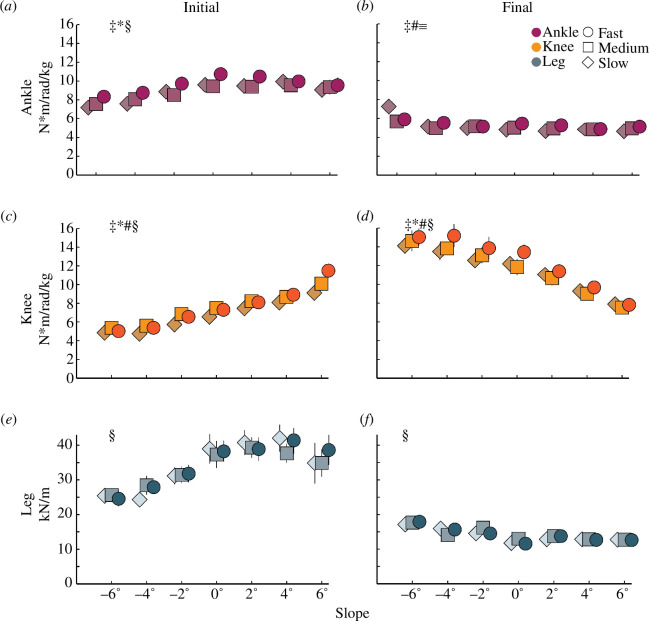
Average ankle (*a*,*d*) and knee (*b*,*e*) joint quasi-stiffness and theoretical leg stiffness (*c*,*f*) during the initial (*a–c*) and rebound joint quasi-stiffness and rebound theoretical leg stiffness during the final (*d–f*) half of the stance phase when subjects ran at three speeds (slow, medium and fast) on seven slopes (±6°, ±4°, ±2° and 0°). Slow, medium and fast speeds for each slope are listed in table 1. Symbols for speeds are offset at each slope for clarity: ‡, significant main effect of speed when running on uphill slopes; *, significant main effect of speed when running on downhill slopes; #, significant main effect of uphill slope; §, significant main effect of downhill slope; ≡, significant interaction between speed and downhill slope (*p* < 0.05). Error bars indicate standard error and may not be seen behind the symbols.

**Figure 3.**
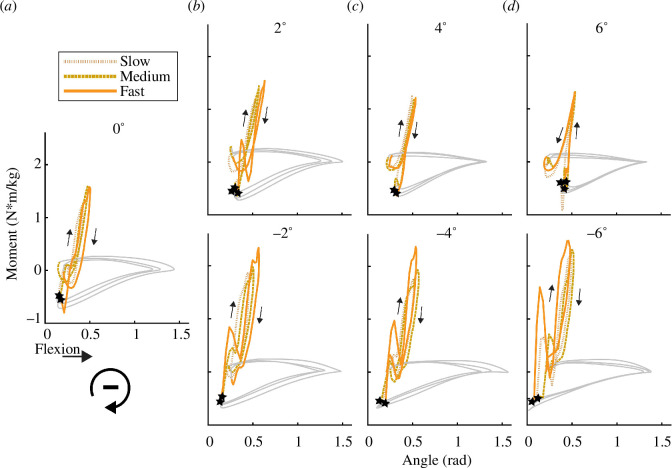
Knee joint moment versus angle for a step during running at (*a*) 0°, (*b*) ±2°, (*c*) ±4° and (*d*) ±6° at slow, medium and fast speeds (table 1) for a representative subject. The slope of the line (quotient of change in moment and change in angle) from 20% of the peak joint moment (*M*_peak_) to maximum joint angular displacement represents initial *k*_*kneeI*_ and from maximum joint angular displacement to 20% of *M*_peak_ represents final *k*_*kneeR*_. Positive knee angle indicates flexion. ⋆ indicates foot contact. The clockwise work loop indicates net negative joint mechanical work and the area inside the loop represents the magnitude of work. The grey lines represent swing phase and the coloured lines represent the stance phase for uphill, level and downhill slopes.

**Figure 4.**
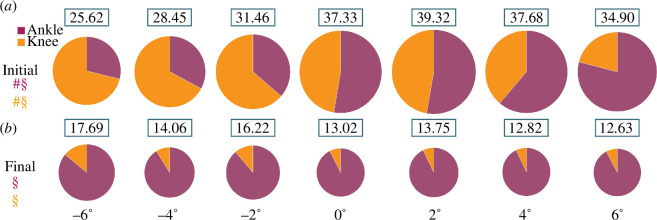
Average ankle (purple) and knee (orange) joint percentage (%) contributions to theoretical leg stiffness and rebound theoretical leg stiffness (kN m^−1^) during the (*a*) initial and (*b*) final half of stance, respectively, at the ‘medium’ speed (table 1) for each slope (0°, ±2°, ±4° and ±6°). We analysed initial and final ankle and knee joint contributions to theoretical leg stiffness at all speeds in our linear mixed-effects models (table 2) but for ease of visualization only present pie charts for one speed at each slope. The area of each circle represents the total theoretical leg stiffness magnitude and the number above each circle is the average theoretical leg stiffness value (kN m^−1^). #, Significant main effect of uphill slope; §, significant main effect of downhill slope (*p* < 0.05) for joint percentage contributions.

In general, when subjects ran at faster speeds, we found that they changed initial and final ankle and knee joint quasi-stiffness and initial ankle and knee joint contributions to theoretical leg stiffness. During the first half of the stance phase, subjects increased their initial *k*_*ankleI*_ when running at faster speeds on uphill (*B* = 0.91 for every 1 m s^−1^ increase in speed, *p* < 0.05; figures 1 and 2*a*) and downhill (*B* = 1.29 for every 1 m s^−1^ increase in speed, *p* < 0.01; figures 1 and 2*a*) slopes and increased initial *k*_*kneeI*_ when running at faster speeds on uphill (*B* = 1.64 for every 1 m s^−1^ increase in speed, *p* < 0.01; figure 2*b* and 3) and downhill (*B* = 0.58, *p* < 0.05; figures 2*b* and 3) slopes. During the second half of the stance phase, subjects increased final *k*_*ankleR*_ when running at faster speeds on uphill slopes (*B* = 0.68 for every 1 m s^−1^ increase in speed, *p* < 0.01; figures 1 and 2*d*) and increased final *k*_*kneeR*_ when running at faster speeds on uphill (*B* = 0.86 for every 1 m s^−1^ increase in speed, *p* < 0.05; figures 2*e* and 3) and downhill (*B* = 2.01 for every 1 m s^−1^ increase in speed, *p* < 0.01; figures 2*e* and 3) slopes. Subjects also decreased the initial ankle joint contribution and increased the initial knee joint contribution (*B* = ±7.67 for every 1 m s^−1^ increase in speed, *p* < 0.05; figure 4) when running at faster speeds on uphill slopes. We did not detect any other statistically significant main effects of speed.

**Table 2.** Average ±s.e.m. theoretical leg stiffness (k_legT_) values and ankle (R_ankle_^2^/*k*_*ankle*_) and knee (R_knee_^2^/*k*_*knee*_) joint contributions (equation (2.3)) to k_legT_ during the first and second half of stance (initial and final, respectively) during running at the medium speed on each slope.

		slope						
		−6°	−4°	−2°	0°	+2°	+4°	+6°
initial	k_legT_ (kN m^−1^)	25.6 ± 2.0	28.5 ± 2.7	31.5 ± 2.4	37.3 ± 3.9	39.3 ± 2.9	37.7 ± 2.7	34.9 ± 3.9
	ankle (%)	28.8 ± 3.7	32.9 ± 4.1	36.4 ± 4.4	52.7 ± 4.2	52.8 ± 3.7	61.2 ± 4.5	79.0 ± 3.4
	knee (%)	71.2 ± 3.7	67.1 ± 4.1	63.6 ± 4.4	47.3 ± 4.2	47.2 ± 3.7	38.8 ± 4.5	21.0 ± 3.4
final	k_legT_ (kN m^−1^)	17.7 ± 1.4	14.1 ± 1.0	16.2 ± 1.4	13.0 ± 1.1	13.8 ± 1.1	12.8 ± 1.1	12.6 ± 1.0
	ankle (%)	86.1 ± 2.2	91.1 ± 0.8	88.8 ± 1.3	92.5 ± 1.0	92.9 ± 0.7	93.0 ± 0.9	92.3 ± 1.3
	knee (%)	13.9 ± 2.2	8.9 ± 0.8	11.2 ± 1.3	7.5 ± 1.0	7.1 ± 0.7	7.0 ± 0.9	7.7 ± 1.3

### 3.2. Main effect of uphill slopes

In general, when subjects ran on steeper uphill slopes regardless of speed, we found that they changed initial knee joint quasi-stiffness, final ankle and knee joint quasi-stiffness, and the initial ankle and knee joint contributions to theoretical leg stiffness. During the first half of the stance phase, subjects increased initial *k*_*kneeI*_ (*B* = 0.76 for every 1° increase in slope, *p* < 0.01; figures 2*b* and 3) on steeper uphill slopes. During the second half of the stance phase, subjects increased final *k*_*ankleR*_ on steeper uphill slopes (*B* = 0.07 for every 1° increase in slope, *p* < 0.05; figures 1 and 2*d*) and decreased final *k*_*kneeR*_ (*B* = −0.60 for every 1° increase in slope, *p* < 0.01; figures 2 and 3*e*). Subjects also increased the initial ankle joint contribution and decreased the initial knee joint contribution (*B* = ±2.90 for every 1° increase in slope, *p* < 0.01; figure 4) to k_legTI_ on steeper uphill slopes. We found no other statistically significant main effects of uphill slope.

### 3.3. Main effect of downhill slopes

In general, when subjects ran on steeper downhill slopes regardless of speed, we found that they changed initial ankle and knee joint quasi-stiffness and theoretical leg stiffness, final knee joint quasi-stiffness and theoretical leg stiffness, and initial and final ankle and knee joint contributions to theoretical leg stiffness. During the first half of the stance phase, subjects decreased initial *k*_*ankleI*_ (*B* = −0.37 for every 1° decrease in slope, *p* < 0.01; figures 1 and 2*a*), decreased initial *k*_*kneeI*_ (*B* = −0.34 for every 1° decrease in slope, *p* < 0.01; figures 2*b* and 3) and decreased initial k_legTI_ (*B* = −2.03 for every 1° decrease in slope, *p* < 0.01; figure 2*c*) on steeper downhill slopes. During the second half of the stance phase, subjects increased final *k*_*kneeR*_ (*B* = 0.42 for every 1° decrease in slope, *p* < 0.01; figures 2*e* and 3*e*) and increased final k_legTR_ (*B* = 0.80 for every 1° decrease in slope, *p* < 0.01; figure 2*f*) on steeper downhill slopes. Subjects also decreased the initial ankle joint contribution and increased the initial knee joint contribution (*B* = ±3.68 for every 1° decrease in slope, *p* < 0.01; figure 4), and decreased the final ankle joint contribution and increased the final knee joint contribution (*B* = ±0.86 for every 1° decrease in slope, *p* < 0.01; figure 4) to k_legTR_ on steeper downhill slopes. We found no other statistically significant main effects of downhill slope.

### 3.4. Interaction effect of speed and downhill slope

We found a significant interaction between speed and downhill slope. During the second half of the stance phase, subjects increased final *k*_*ankleR*_ (*B* = 0.33, *p* < 0.01; figures 1 and 2*d*) when running on steeper downhill slopes at faster speeds where this effect was different at the −6° slope where *k*_*ankleR*_ decreased at faster running speeds. We found no other statistically significant interaction effects of speed and downhill slope.

## 4. Discussion

### 4.1. Speed

We partially accept our first hypothesis because we found that at faster speeds, runners increased initial ankle and knee joint quasi-stiffness during the first half of the stance phase on uphill and downhill slopes and increased final ankle rebound joint quasi-stiffness on uphill and final knee rebound joint quasi-stiffness on uphill and downhill slopes during the second half of the stance phase. We also found that initial ankle and knee joint contributions to theoretical leg stiffness decreased and increased, respectively, at faster speeds but only when running on uphill slopes. We did not detect any significant differences in initial theoretical leg stiffness or final rebound theoretical leg stiffness across the measured speeds, which is in contrast with McGowan *et al*. [28]. Our results may differ from those of McGowan *et al*. owing to the different speed ranges between studies, where McGowan *et al*. measured leg stiffness during running from 3 m s^−1^ to maximum sprinting speed and found that leg stiffness primarily increased at speeds faster than 6 m s^−1^. At speeds faster than 6 m s^−1^, leg length displacement does not change but peak vertical GRF increases, which results in greater leg stiffness [28]. Previous studies and the present study found that at slow to moderate speeds, leg stiffness does not change because leg length displacement and peak vertical GRF change proportionally [29,30]. Our results are in line with those of Jin & Hahn [14] who found that ankle and knee joint quasi-stiffness increased by ~28 and ~22% (for a ~1.0 m s^−1^ increase in speed), respectively, during the first half of stance when running at 3.0 m s^−1^ compared with 2.0 m s^−1^ on level ground. Based on the linear mixed model regressions, we found that ankle joint quasi-stiffness increased by ~17% for a 1.0 m s^−1^ change in speed and knee joint quasi-stiffness increased by ~22% for a 1.0 m s^−1^ increase in speed during the first half of stance regardless of slope.

Our findings can be used to inform passive-elastic device designs that are capable of exhibiting variable stiffness throughout the stance phase [21]. Our results suggest that a passive-elastic assistive device designed to mimic a biological ankle and knee joint such as a running-specific prosthesis (RSP) for a recreational runner with a transfemoral amputation who runs at 1.5–3.0 m s^−1^ should incorporate a change in stiffness within the prosthetic blade (the lower portion of an RSP consists of carbon fibre) and prosthetic knee joint. When running at faster speeds regardless of slope, the prosthetic blade and knee joint should stiffen during the first half of stance and the prosthetic knee joint should stiffen during the second half of stance. When running faster on uphill slopes, the prosthetic blade should also stiffen during the second half of stance. Moreover, the contribution of the prosthetic blade and knee to overall leg stiffness should decrease and increase, respectively, during the first half of the stance phase to enable a faster running speed on level ground and uphill slopes. Because the stiffness of currently available prosthetic blades cannot be changed dynamically, changes in the blade stiffness could be accomplished by changing the alignment of the RSP relative to the GRF vector [31] and changes to the prosthetic knee stiffness could be achieved by changing the resistance within the prosthetic knee joint and/or the maximum prosthetic knee joint angle. Despite the complexities of achieving a specific joint external moment arm or GRF vector, to accomplish the joint contribution changes, future device designs should allow dynamic changes in external moment arms and stiffness. Additionally, an individual with a transfemoral amputation may be able to change their running biomechanics to decrease the external moment arm about the prosthetic blade and increase the external moment arm about the prosthetic knee joint to decrease and increase the contribution, respectively, to leg stiffness.

### 4.2. Uphill slopes

We partially accept our second hypothesis because we found that subjects increased initial knee joint quasi-stiffness during the first half of stance and increased final ankle rebound joint quasi-stiffness during the second half of stance to run on steeper uphill slopes up to +6° compared with level ground. However, contrary to our hypothesis, decreased final knee rebound joint quasi-stiffness during the second half of stance to run on steeper uphill slopes up to +6° compared with level ground. We also found that subjects increased the initial ankle joint contribution and decreased the initial knee joint contribution to theoretical leg stiffness. Our results are consistent with Khassetarash *et al*. [16], who found that when running +5° uphill at 3.3 m s^−1^, knee joint quasi-stiffness during the first half of the stance phase increased to ~343 N m rad^−1^ compared with ~229 N m rad^−1^ on level ground [16]. We found that during the first half of the stance phase, knee joint quasi-stiffness increased by 0.76 N m rad^−1^ kg^−1^ for every 1° increase in slope. At the medium running speed, initial knee joint quasi-stiffness increased from 7.49 N m rad^−1^ kg^−1^ on level ground to 10.1 N m rad^−1^ kg^−1^ at +6°. Additionally, our results are partially consistent with those of Nuckols *et al*. [18], who found that the muscles surrounding the ankle joint contributed the most positive mechanical power (47–55%) compared with the knee and hip joints when running uphill at 2.86° and 5.71° at 2.25 m s^−1^. We found that during the first half of stance at +4° and +6° the ankle joint contribution to theoretical leg stiffness was 62.7 and 79.0%, respectively, but during the second half of stance at +2°, +4° and +6°, the ankle joint contribution to rebound theoretical leg stiffness was 92.3–93.0%. Our results may differ because we did not include the hip joint in our calculations and Nuckols *et al*. [18] found that the hip joint positive mechanical power contribution was 34 and 36% of the total positive mechanical power during running at 2.25 m s^−1^ on 2.86° and 5.71° slopes, respectively. Furthermore, Nuckols *et al*. [18] investigated net mechanical power while we calculated the joint contributions to theoretical leg stiffness.

Our results suggest that a passive-elastic assistive device designed to mimic a biological ankle and knee joint such as an RSP for an individual with a transfemoral amputation should not change overall stiffness during the stance phase to allow running on steeper uphill slopes but should allow dynamic changes within the prosthetic blade and prosthetic knee. When running on steeper uphill slopes, the prosthetic knee joint should stiffen during the first half of stance, and the blade should stiffen, and prosthetic knee joint should become less stiff during the second half of the stance phase. Moreover, the contribution of the prosthetic blade and prosthetic knee to overall leg stiffness should increase and decrease during the first half of stance, respectively, when running on steeper uphill slopes compared with level ground. Despite the complex interactions of achieving a given external moment arm and GRF vector, to accomplish these contribution changes, future device designs should allow dynamic changes in external moment arms and stiffness. Additionally, an individual with a transfemoral amputation may be able to change their biomechanics to increase the external moment arm about the prosthetic blade and decrease the external moment arm about the prosthetic knee joint to increase and decrease the contribution, respectively, to leg stiffness. This could also be accomplished by manipulating RSP alignment and/or prosthetic knee joint angles dynamically. For example, by reducing flexion at the prosthetic knee joint during the first half of stance, the external moment arm could be reduced and the contribution of the prosthetic knee joint to theoretical leg stiffness would decrease (equation (2.3)).

### 4.3. Downhill slopes

We partially accept our third hypothesis. In support of our hypothesis, when running on steeper downhill slopes we found that subjects decreased initial ankle joint quasi-stiffness and theoretical leg stiffness during the first half of stance. Contrary to our hypothesis, when running on steeper downhill slopes we found that subjects decreased initial knee joint quasi-stiffness during the first half of the stance phase and increased final knee rebound joint quasi-stiffness and theoretical leg stiffness during the second half of stance. We also found that in support of our hypothesis, when running on steeper downhill slopes down to −6° compared with level ground, the contribution of the ankle joint and knee joint to theoretical leg stiffness decreased and increased, respectively, during the first half of the stance phase. Subjects also decreased the final ankle joint contribution to leg stiffness and increased the final knee joint contribution to rebound theoretical leg stiffness during the second half of stance. Our results are consistent with Khassetarash *et al*. [16], who found that when running −5° downhill at 3.3 m s^−1^, ankle joint quasi-stiffness during the first half of the stance phase decreased to ~286 N m rad^−1^ compared with ~401 N m rad^−1^ on level ground [16]. We found that during the first half of the stance phase, ankle joint quasi-stiffness decreased by −0.37 N m rad^−1^ kg^−1^ for every 1° decrease in slope. At the medium running speed, initial ankle joint quasi-stiffness decreased from 9.4 N m rad^−1^ kg^−1^ on level ground to 7.5 N m rad^−1^ kg^−1^ at −6°. Additionally, our results are partially consistent with those of Nuckols *et al*. [18], who found that the ankle contributed the most positive net mechanical power (47–55%) compared with the knee and hip when running downhill at −5.71° and −2.86° at 2.25 m s^−1^. We found that during the first half of stance at −2, −4° and −6° the knee joint contributed 63.6, 67.1 and 71.2%, respectively, to theoretical leg stiffness but during the second half of stance at −2°, −4° and −6°, the ankle joint contributed 86.1–91.1% to rebound theoretical leg stiffness. Our results may differ because we did not include the hip joint in our calculations and Nuckols *et al*. [18] investigated net mechanical power while we calculated the ankle and knee joint contributions to theoretical leg stiffness.

Our results suggest that a passive-elastic assistive device designed to mimic a biological ankle and knee joint such as an RSP for an individual with a transfemoral amputation should decrease overall stiffness during the first half of the stance phase and increase overall stiffness during the second half of the stance phase when running on steeper downhill slopes compared with level ground and allow changes within the prosthetic blade and prosthetic knee joint. When running on steeper downhill slopes, the prosthetic blade and the prosthetic knee should decrease stiffness during the first half of stance, but the prosthetic knee should stiffen during the second half of the stance phase. Moreover, the contribution of the prosthetic blade and prosthetic knee to overall leg stiffness should decrease and increase, respectively, during the first and second half of stance. Despite the complexities of achieving a specific external moment arm or GRF vector, to accomplish these contribution changes a device should allow dynamic external moment arm and stiffness changes. Additionally, an individual with a transfemoral amputation could decrease the external moment arm about the prosthetic blade and increase the external moment arm about the prosthetic knee joint. This could be accomplished by manipulating RSP alignment and/or prosthetic knee joint angles. For example, by increasing flexion at the prosthetic knee during stance, the external moment arm could be increased and the contribution of the knee joint to theoretical leg stiffness would increase (equation (2.3)).

### 4.4. Interaction between speed and downhill slope

We found a significant interaction effect between speed and downhill slope. When running on steeper downhill slopes, subjects increased final ankle rebound joint quasi-stiffness. On 0°, −2° and −4° slopes, final ankle rebound joint quasi-stiffness also increased with speed but on the −6° slope, final ankle rebound joint quasi-stiffness decreased with speed. These results suggest that a passive-elastic assistive device designed to mimic a biological ankle such as an RSP should allow changes within the prosthetic blade that depend on the downhill slope. When running on steeper downhill slopes up to −4°, the prosthetic blade should increase stiffness during the first half of stance and increase further as running speed increases. When running on −6° slopes and perhaps even steeper, the prosthetic blade should become less stiff as running speed increases. Our results indicate that ankle and knee joint quasi-stiffness and joint contributions to theoretical leg stiffness are adjusted when running at different speeds and uphill and downhill slopes, information important for biomimetic device design. For example, future studies and design ideas for prosthetic devices should consider the stiffness and alignment of an RSP with respect to the socket and residual limb and with respect to the GRF vector [31]. In addition, prosthetic blades do not articulate like a biological ankle and are primarily designed to incorporate a forefoot strike pattern, which may not be ideally suited to mimic a biological ankle for long distance and downhill running. Some prosthetic blades incorporate a heel component, which athletes may choose for downhill running. The incorporation of a heel component could change the prosthetic blade stiffness during the first half of the stance phase, thus future studies are needed to quantify prosthetic blade stiffness changes that occur during running on level ground and slopes with different alignments and with or without a heel component. To mimic the biological ankle, prosthetic blades should incorporate the range of motion (articulate) for uphill and downhill running. To mimic a biological knee for individuals with a transfemoral amputation, a prosthetic knee should also be articulated. The range of motion and limb segment position of the ankle and knee joints can change the external moment arm and the effective mechanical advantage (EMA) of the leg, where EMA is defined as the quotient of the internal moment arm and external moment arm length. A greater EMA results in a lower metabolic cost, which would improve distance running performance [32]. Future studies are also warranted to investigate how hip joint mechanics affect overall leg stiffness, especially when running uphill. Previous studies have found that hip joint net positive mechanical power increases with the uphill slope and its contribution to total positive power increases from 28% on level ground to 36% at 5.71° [18]. We did not incorporate hip joint external moment arm and stiffness into our calculations of theoretical leg stiffness because peak hip joint moment and angular displacement did not occur within 10% of mid-stance, and the hip joint primarily extends and does not behave like a torsional spring during the stance phase of running on level, uphill and downhill slopes (electronic supplementary material, figure S2 and S3).

Overall, ankle and knee joint quasi-stiffness and rebound joint quasi-stiffness during the first and second half of stance, respectively (figures 1 and 3), provide a framework for informing passive-elastic biomimetic device designs such as footwear, which acts in series with the leg, lower limb exoskeletons, which act in parallel with the leg, and running-specific leg prostheses, which act in series with the residual limb. Ankle and knee joint quasi-stiffness also allows comparisons between different device configurations that may incorporate different stiffness profiles in series or in parallel with the legs [31]. Future studies are needed to determine how a device with variable quasi-stiffness affects running biomechanics and metabolic costs [33]. Furthermore, device designs that detect changes in speed and slope may be needed to enable a biomimetic response. While we did not statistically compare joint quasi-stiffness during the first half of the stance phase to rebound joint quasi-stiffness during the second half of stance, these values appear to differ at the ankle and knee (figure 2). This indicates that the joints may not behave like perfect springs when running on uphill and downhill slopes. However, passive-elastic device designs rely on stiffness values to provide a spring-like function to the user. Thus, we determined joint quasi-stiffness to inform these device designs.

There are a few potential limitations of the study. We asked subjects to run at slower speeds for steeper uphill slopes than for level ground and downhill slopes, with a range of 1.5–3.0 m s^−1^. Thus, our results may not be applicable to faster speeds and future research is needed to understand speed-specific joint and leg stiffness changes when runners navigate slopes. Owing to the lack of overlapping speeds on uphill slopes, we did not determine if there were interaction effects between speed and uphill slope. Thus, future research is needed to determine if there are interactions between speed and uphill slope on joint and leg stiffness. Furthermore, we calculated leg stiffness from ankle and knee joint external moment arms and quasi-stiffness. It is possible that there are inter-joint interactions within the leg that may affect overall leg stiffness and may not be accounted for within the equation used to calculate theoretical leg stiffness [9]. Moreover, the hip likely contributes to overall leg stiffness, especially at steeper uphill slopes owing to a greater external moment arm (table 1), but this contribution is not accounted for in the equation that Moholkar derived to calculate theoretical leg stiffness as maximum hip joint angular displacement does not occur within 10% of mid-stance, but ankle and knee joint angular displacement do occur within 10% of mid-stance [9,19]. We chose to compare discrete stiffness values to describe ankle and knee joint behaviour during uphill, downhill and level running, and used these discrete values to determine theoretical leg stiffness (equations (2.2) and (2.3)). However, the lower extremities’ muscular, tendinous, ligamentous and skeletal structures may not operate as purely passive-elastic systems with discrete stiffness values for the first and second half of the stance phase, which may limit the application of the results. Thus, future studies that compare the relationship between joint moment and joint angle throughout the stance phase (figures 1 and 3) may be more precise and informative for the characterization of the contributions of the ankle and knee joints to overall theoretical leg stiffness when running on slopes and could be used to more specifically inform the development of assistive devices that could modulate stiffness dynamically during the stance phase.

Future experimental and musculoskeletal modelling studies are warranted to determine how the stiffness and user–device interface of device designs that span one joint or multiple joints affect inter-joint interactions and overall leg stiffness. Additionally, hip joint quasi-stiffness and the contribution of the hip joint to overall leg stiffness when running on slopes should be investigated and considered for future device design. Future research is also warranted to compare the biomechanical effects of assistive devices on biological joints during running on slopes at different speeds. While variable stiffness quasi-passive prostheses have been designed and experimentally tested for walking, further research and development are needed to design and experimentally test variable stiffness quasi-passive assistive devices for running at different speeds and on slopes [21–23]. Moreover, manipulating the joint and leg stiffness of a device can be used to determine whether such changes improve biomechanical asymmetry and/or the metabolic cost of running on slopes at different speeds.

## 5. Conclusion

We determined ankle and knee joint quasi-stiffness and theoretical leg stiffness of humans running at different speeds (1.5–3.0 m s^−1^) on level, uphill and downhill slopes (0°, ±2°, ±4° and ±6°) to inform lower limb device designs. We used a previously derived equation [9] to calculate theoretical leg stiffness from ankle and knee joint external moment arms and stiffness to determine the contributions to overall leg stiffness during running at different speeds on level, uphill and downhill slopes. These findings can be used for passive-elastic device designs, and specifically for footwear, exoskeletons and running-specific leg prostheses that aim to mimic and/or enhance the biological ankle and/or knee joints. Further research is needed to determine how the stiffness of a device affects leg stiffness and performance during running at different speeds and on uphill and downhill slopes.

## Data Availability

The authors confirm that all data underlying the findings are fully available without restriction. Electronic supplementary material is available online [34].
